# Empowering sexual self-care in HPV-positive women: A randomized trial of health belief model–Based education

**DOI:** 10.1371/journal.pone.0338192

**Published:** 2025-12-11

**Authors:** Sanaz Akhondzadeh, Zahra Behboodi Moghadam, Masoumeh Namazi, Shima Haghani, Akram Ghahghaei Nezamabadi

**Affiliations:** 1 Department of Midwifery and Reproductive Health, School of Nursing and Midwifery, Tehran University of Medical Sciences, Tehran, Iran; 2 Department of Midwifery and Reproductive Health, School of Nursing and Midwifery, Tehran University of Medical Sciences, Tehran, Iran; 3 Department of Midwifery and Reproductive Health, School of Nursing and Midwifery, Tehran University of Medical Sciences, Tehran, Iran; 4 Nursing and Midwifery Care Research Center, Health Management Research Institute, Iran University of Medical Sciences, Tehran, Iran; 5 Department of Obstetrics and Gynecology, School of Medicine, Tehran University of Medical Sciences, Tehran, Iran; Johns Hopkins University School of Medicine, UNITED STATES OF AMERICA

## Abstract

**Introduction:**

The human papillomavirus (HPV) is the most common sexually transmitted infection worldwide. The present study aimed to examine the impact of an educational intervention based on the Health Belief Model (HBM) on sexual self-care among women of reproductive age infected with HPV.

**Method:**

This study was a randomized controlled trial with two parallel groups. Seventy-two women with HPV were selected through simple random sampling from Arash and Imam Khomeini hospitals in Tehran, Iran, and were allocated to a control group (n = 35) and an intervention group (n = 37). Participants in the intervention group received Health Belief Model (HBM)-based sexual self-care education during four weekly sessions, each lasting 45–60 minutes. The sessions were conducted using lectures, group discussions, question-and-answer sessions, PowerPoint presentations, pamphlets, educational videos, and role-playing. Data were collected using a demographic and obstetric information questionnaire, the Sexual Self-Care Questionnaire, and the HBM Questionnaire at three stages: before the intervention, immediately after, and 8 weeks after the completion of the intervention. Data analysis was performed using independent t-tests, Fisher’s exact test, chi-square test, and repeated measures ANOVA, with SPSS version 26 software.

**Results:**

Both groups participating in the study were homogeneous in terms of demographic and obstetric characteristics (p > 0.05). The mean total score of sexual self-care and all its dimensions did not show a statistically significant difference between the intervention and control groups before the intervention (p = 0.275). However, immediately after and 8 weeks after the completion of the intervention, a significant increase was observed in the intervention group (p < 0.001). Additionally, the implementation of the intervention in the intervention group led to a significant increase in the constructs of perceived sensitivity, perceived benefits, cues to action, and self-efficacy, as well as a significant decrease in perceived barriers, both immediately and 8 weeks after the completion of the intervention (p < 0.001). However, the construct of perceived severity regarding sexual self-care did not show a statistically significant difference between the two groups at any time point (p > 0.05).

**Conclusion:**

Based on the results of the study, education based on the Health Belief Model has been effective in improving sexual self-care and enhancing the constructs of the model in women with human papillomavirus (HPV). Therefore, it is recommended that this theory be considered by healthcare providers and midwives when educating patients with human papillomavirus.

**Trial registration:**

This study was registered in the Iranian Registry of Clinical Trials on 2024/01/01 (code: IRCT20231223060503N1)

URL: https://irct.behdasht.gov.ir/trial/74692

## Introduction

The human papillomavirus (HPV) is the most common sexually transmitted infection worldwide [1]. HPV strains are classified into low-risk and high-risk types based on their association with cancer [2]. The global prevalence of HPV is estimated to be 11.7%, with the highest rates observed in Southern Africa (17.4%), Eastern Africa (33.6%), Eastern Europe (21.4%), and Western Europe (9.0%) [3]. HPV transmission occurs through direct skin-to-skin or mucosal contact during vaginal, anal, or oral sex. Both asymptomatic and symptomatic individuals infected with HPV can transmit the virus [4]. However, most anogenital HPV infections resolve on their own; for example, over 90% of cervical infections clear within 1–3 years [5]. High-risk sexual behaviors, including a young age at first vaginal or oral intercourse and having more sexual partners, are the main risk factors associated with the acquisition and persistence of HPV infection and the development of related cancers. These sexual risk factors vary based on socioeconomic status, age, race, and education level [6,7]. It is estimated that around 2.8% of Iranian women in the general population have HPV16/18 infection. Iran has a population of about 33.5 million women aged 15 years and older at risk of developing cervical cancer, with 1,056 women diagnosed with cervical cancer and 644 deaths annually [8].

Most HPV-related diseases are preventable through education, screening programs, and vaccination [1]. HPV infection and genital warts have significant physical and psychological effects on women [9]. The more severe the disease, the greater the impact on mental health, which also imposes financial costs on healthcare systems [10]. A lack of awareness about safe sexual practices, the protective benefits of HPV vaccination, and the need for a range of tests could be the primary reasons for these issues [11].

Self-care refers to the ability of individuals, families, and communities to take intentional and purposeful actions to promote and maintain their health, prevent illness, and manage disease and disability, with or without the support of healthcare providers [12]. Effective techniques for enhancing self-care behaviors in women are organized into three categories: lifestyle modification, preventive behaviors and screening, and disease and treatment management behaviors. Self-care behaviors that help prevent HPV infection include undergoing Pap smears, vaccination, consistent and correct use of condoms during sexual intercourse, and limiting sexual partners to one [11,12]. Self-care interventions are among the most important and promising approaches to improving universal health coverage and well-being, both for health systems and individuals. As accessible, cost-effective, and acceptable interventions, they promote self-efficacy, independence, and participation in health [13], yet nearly four billion women of reproductive age worldwide lack access to such services [14].

One of the most widely used theories in health behavior is the Health Belief Model (HBM), which has extensive applications in health education for various health-related issues. The HBM posits that six constructs predict health behavior: perceived susceptibility, perceived severity, perceived benefits, perceived barriers, self-efficacy, and cues to action [15]. A study by Bayrami et al. (2019) aimed to assess the constructs of the HBM in relation to the willingness to receive HPV vaccination among female students at Urmia University of Medical Sciences. According to the results, perceived susceptibility, perceived severity, perceived benefits, perceived barriers, self-efficacy, and cues to action were all significantly associated with the acceptance of the HPV vaccine [16].

According to the results of a qualitative study conducted in Iran (2023), most women infected with HPV lacked sufficient knowledge about the virus, including its causes, symptoms, complications, prevention, and screening methods. Education, counseling, support, and healthcare services were identified as the main needs and challenges in reproductive and sexual health [17]. Based on the literature review, most studies have focused on the prevention of HPV infection. However, individuals who are already affected by this condition face numerous challenges, highlighting the need for further research into self-care practices for this group of patients. Given the Iranian and Islamic cultural context, HPV infection may be associated with negative psychological effects and significant challenges for Iranian women due to its sexual nature and the stigma attached to it, complicating efforts to educate, support, and address their needs [18,19]. Therefore, the present study aimed to examine the impact of an educational intervention based on the Health Belief Model (HBM) on sexual self-care among married women of reproductive-age infected with HPV.

## Method

### Patients and study design

**Fig 1:**
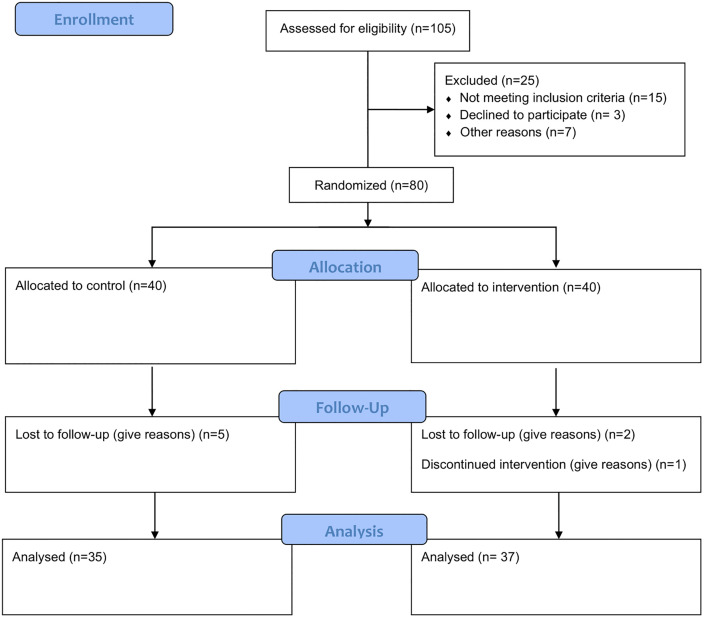
Participants’ flow in the study.

This randomized clinical trial was conducted from April to December 2024 at Arash Women’s Comprehensive Hospital and Imam Khomeini Hospital, two referral centers in Tehran, Iran. The study population consisted of women with a confirmed HPV-DNA test who attended gynecology clinics at these hospitals. The trial adhered to the CONSORT guidelines (Fig 1) [20].

Participants were eligible if they were Iranian, married, between 15 and 49 years of age, literate (able to read and write), and had no history of chronic psychiatric disorders, malignant diseases, or previous participation in educational or counseling sessions related to sexually transmitted infections (STIs). Women were excluded if they were absent from more than two educational sessions, submitted incomplete or incorrect questionnaires, or expressed unwillingness to continue participation.

After obtaining approval from the Ethics Committee of Tehran University of Medical Sciences (IR.TUMS.FNM.REC.1402.186) on 2023/12/19 and registering the trial (IRCT20231223060503N1) on 2024/01/01, eligible participants were recruited through convenience sampling. Written informed consent was obtained from all women, and those who met the criteria were randomly allocated to either the intervention or control group using block randomization (block size = 4) with a computer-generated sequence.

Allocation concealment was maintained using opaque, sequentially numbered envelopes, and outcome assessors were blinded to group assignments. Although blinding of researchers and participants was not feasible due to the nature of the intervention, the outcome evaluator remained blinded by having a separate colleague handle data collection.

The intervention group attended four weekly, face-to-face educational sessions (45–60 minutes each) based on the constructs of the Health Belief Model (HBM). Sessions were delivered in groups of 10–15 participants by the researcher and employed interactive methods including lectures, group discussions, Q&A, PowerPoint presentations, pamphlets, videos, and role-playing (Table 1). The control group received routine care during the study period. Questionnaires were administered to both groups at baseline, immediately after the intervention, and eight weeks post-intervention. Participants in the control group received routine care as provided by the hospitals’ gynecology clinics, which typically included standard counseling on reproductive health, general advice on hygiene, and routine check-ups. Participants continued to receive care as they normally would, without additional structured education. The educational content delivered to the intervention group did not overlap with routine care topics, ensuring that observed effects could be attributed to the HBM-based educational sessions. To uphold ethical considerations, control group participants were offered educational pamphlets and CDs after the study concluded if they expressed interest.

**Table 1:** Health Belief Model-Based Educational Intervention.

Session	Main Objective	Educational Content	Teaching Methods and Schedule
First	Improve awareness about HPV and sexual self-care	PowerPoint presentation, Session 1 handout	1) Introduction of the facilitator and participants, study objectives (10 min) 2) Information on HPV, its global and local prevalence, and the importance of sexual self-care (40 min) 3) Q&A session (10 min)
Second	Impact on perceived susceptibility, perceived severity, and perceived benefits of the Health Belief Model	PowerPoint presentation, Session 2 handout	1) Review of the previous session (5 min) 2) Explanation of the consequences of not having a Pap smear test, non-vaccination against non-infecting strains, effects on marital life and quality of life, issues with having multiple sexual partners (45 min) 3) Q&A session to address perceived benefits (10 min)
Third	Impact on perceived barriers and self-efficacy of the Health Belief Model	PowerPoint presentation, Session 3 handout, Educational video	1) Review of previous sessions (5 min) 2) Request women with HPV to share their issues since the onset of symptoms or diagnosis, discuss the concept of sexual self-care and its connection to self-efficacy (40 min) 3) Share common experiences and solutions with each other, summarize the taught materials using the educational video (15 min) 4) Q&A session (10 min)
Fourth	Impact on cues to action and performance of the Health Belief Model	PowerPoint presentation, Session 4 handout, Educational video, Role-playing	1) Review of previous sessions (5 min) 2) Teach various sources of information about HPV (15 min) 3) Ask women to express their fears about symptoms and provide self-care recommendations, advise appropriate behaviors, and communicate problems and needs to healthcare providers (30 min) 4) Role-playing, educational video, and summarization of the taught materials (10 min) 5) Q&A session (10 min)

### Sample size

The required sample size was calculated using a confidence level of 95%, 80% power, and an expected effect size (Cohen’s d = 0.7) based on previous studies reporting medium to large effects of theory-based sexual health interventions [21,22]. This calculation yielded 32 participants per group; accounting for a 10% dropout rate, 35 participants were enrolled in each group.

### Outcomes

The primary outcome of the study was sexual self-care, measured using the Sexual Self-Care in Women of Reproductive Age (FSHS) questionnaire. The secondary outcomes included participants’ perceived susceptibility, severity, benefits, barriers, and self-efficacy regarding sexual self-care, which were assessed using a researcher-designed questionnaire based on the Health Belief Model (HBM).

### Study instruments

The three study instruments are described below:

- **Demographic and obstetric information questionnaire**: This questionnaire consists of 15 questions, including: age, education level, employment status, income level, duration of marriage, number of pregnancies, number of deliveries, type of delivery, number of abortions, contraceptive method, history of Pap smear, history of HPV vaccination, smoking history, genital warts, and duration of HPV infection based on diagnostic tests.
- **Sexual Self-Care in Women of Reproductive Age (FSHS) questionnaire**: This questionnaire consists of 40 items scored on a 5-point Likert scale ranging from “Never” (1) to “Always” (5). It covers four domains: prevention of sexually transmitted infections (STIs), prevention of gynecological cancers, prevention of unintended pregnancies, and promotion of sexual health. Developed using a deductive approach based on the Waltz model in 2021 by Yazdani and colleagues in Iran, the tool has demonstrated strong psychometric properties. It has a content validity index (CVI) of 0.93, a content validity ratio (CVR) of 0.96, and high internal consistency reliability with a Cronbach’s alpha of 0.94. The instrument’s stability, measured by intra-cluster correlation, was 0.97 (confidence interval: 0.94–0.98), indicating strong validity and reliability in the Iranian population. These properties make it a suitable measure of sexual self-care [23].
- **Researcher-Designed Questionnaire Based on the Health Belief Model**: This questionnaire was designed by the researchers based on the study objectives and relevant literature. The questions are presented in Table 2. To confirm the qualitative content validity of the questionnaire, it was reviewed by 10 faculty members in midwifery and reproductive health at Tehran University of Medical Sciences, and their feedback was incorporated. For the CVR, experts were asked to evaluate each item on a 3-point scale (“essential,” “useful but not essential,” or “not essential”). After calculating the CVR and making necessary revisions, experts also assessed the CVI by rating each item on a scale from 1 to 4 based on relevance (4 = very relevant, 3 = relevant, 2 = somewhat relevant, 1 = not relevant). Face validity was assessed by asking 10 women of reproductive age to provide feedback on the clarity of the questionnaire, and necessary clarifications were made based on their input. Internal consistency was evaluated using Cronbach’s alpha to determine the reliability of the instrument.

**Table 2:** Researcher-Designed Questionnaire Based on the Health Belief Model.

Question Type	Questions	Scale
Perceived Susceptibility	I am at risk of complications from HPV. I am at risk of cervical cancer. I am at risk of high-risk HPV strains. My partner is at risk of the disease.	5-point Likert scale (1 = Strongly disagree, 5 = Strongly agree)
Perceived Severity	The disease causes problems in sexual relations. Thinking about the complications of HPV scares me. Thinking about the problems caused by HPV infection makes me anxious and worried. If I experience HPV complications, my spouse and family will blame me. The cost of treatment is high. Quality of life decreases after contracting the disease.	5-point Likert scale (1 = Strongly disagree, 5 = Strongly agree)
Perceived Benefits	Women who engage in sexual self-care experience fewer physical and emotional crises. Sexual self-care reduces the costs associated with cervical cancer. Sexual self-care prevents my partner from contracting the disease. Sexual self-care allows me to enjoy sexual relations more. Sexual self-care helps prevent high-risk HPV strains.	5-point Likert scale (1 = Strongly disagree, 5 = Strongly agree)
Perceived Barriers	I don’t have enough time to get the HPV vaccine. I don’t have enough time to visit a doctor or midwife. I can’t afford the HPV vaccine. I don’t have access to accurate information about sexual self-care. My partner agrees to use condoms during sexual relations. I feel embarrassed about vaginal exams and Pap smears. I am afraid of vaginal exams and Pap smears. Fear of side effects from the HPV vaccine prevents me from getting it.	5-point Likert scale (1 = Strongly disagree, 5 = Strongly agree)
Cues to Action	Reading brochures and using educational videos are effective for sexual self-care. Media (TV and social media) are effective for sexual self-care. My doctor and midwife assist me with sexual self-care.	5-point Likert scale (1 = Strongly disagree, 5 = Strongly agree)
Self-Efficacy	I can do sexual self-care.	5-point Likert scale (1 = Strongly disagree, 5 = Strongly agree)

### Statistical analysis

Analyses were conducted using linear mixed-effects regression models to account for repeated measures and assess intervention effects over time. Effect estimates (mean differences) with 95% confidence intervals (CI) were reported alongside Cohen’s d for standardized effect sizes. Between-group comparisons at each time point used independent t-tests, and within-group changes over time were assessed using repeated-measures ANOVA. Missing data were minimal (<5%) and handled using complete-case analysis. Statistical significance for secondary outcomes was interpreted conservatively, considering multiple testing and risk of Type I error, with emphasis on primary outcomes.

## Results

The results indicated no significant differences between the control and intervention groups in terms of demographic and obstetric variables. Independent-sample t-test results revealed no significant differences between the intervention and control groups regarding the mean age and duration of marriage (P > 0.05). Fisher’s Exact Test also showed no significant differences between the two groups in terms of education level, employment status, number of abortions, and contraceptive method (P > 0.05). Additionally, chi-square tests demonstrated no significant differences between the groups in economic status, smoking history, number of pregnancies, number of deliveries, type of delivery, history of HPV vaccination, duration of HPV infection, and presence of genital warts (P > 0.05) (Table 3).

**Table 3:** Demographic and Obstetric Characteristics in the Intervention and Control Groups.

Variable	Intervention group		Control group		P-value
	Frequency	Percentage	Frequency	Percentage	
Age (Years)					0.68
Under 30	13	35.1%	10	28.6%	
30–39	10	27%	17	48.6%	
40 and above	14	37.8%	8	22.9%	
Mean ± Standard Deviation	33.8 ± 4.77		34.6 ± 5.83		
Range (Min–Max)	19–48		20–48		
Education Level					0.809
Below Diploma	3	8.1%	4	11.4%	
Diploma	18	48.7%	14	40.1%	
Bachelor’s Degree	12	32.4%	11	31.4%	
Master’s Degree and above	4	10.8%	6	17.1%	
Employment Status					0.847
Housewife	27	73%	28	80%	
Employed	10	27%	7	20%	
Economic status					0.327
Poor	10	27%	7	20%	
Medium	20	54.1%	16	45.7%	
Good	7	18.9%	12	34.3%	
Smoking History					0.681
No	27	73%	24	68.6%	
Yes	10	27%	11	31.4%	
Marriage Duration (Years)					0.705
Less than 5	18	48.6%	8	22.9%	
5–9	2	5.4%	8	22.9%	
10–14	8	21.6%	11	31.4%	
15 and above	9	24.3%	8	22.9%	
Mean ± Standard Deviation	9.8 ± 3.74		10.6 ± 8.89		
Range (Min–Max)	1–29		1–28		
Number of Pregnancies					0.768
0	19	51.4%	19	54.3%	
1	8	21.6%	4	11.4%	
2 or more	10	27%	12	34.3%	
Number of Deliveries					0.977
0	21	56.8%	20	57.1%	
1	7	18.9%	6	17.1%	
2 or more	9	24.3%	9	25.7%	
Type of Delivery					0.875
Vaginal	27	73%	27	77.1%	
Cesarean	10	27%	8	22.9%	
Number of Abortions					0.791
0	31	83.8%	30	85.7%	
1	4	10.8%	2	5.7%	
2 or more	2	5.4%	3	8.6%	
Contraceptive Method					0.307
No method	18	48.6%	14	40%	
Withdrawal	4	10.8%	7	20%	
Condom	10	27%	11	31.4%	
Sterilization	3	8.1%	0	0%	
Hormonal contraceptives	1	2.7%	0	0%	
IUD	1	2.7%	3	8.6%	
History of Pap Smear					-
Yes	37	100%	35	100%	
No	0	0%	0	0%	
History of HPV Vaccination					0.504
No	10	27%	12	34.3%	
Yes	27	73%	23	65.7%	
Duration of HPV Infection					0.412
Less than 1 month	5	13.5%	6	17.1%	
1–6 months	9	24.3%	14	40%	
6–12 months	7	18.9%	4	11.4%	
More than 12 months	16	43.2%	11	31.4%	
Presence of Genital Warts					0.354
No	15	40.5%	18	51.4%	
Yes	22	59.5%	17	48.6%	

p-values are based on t, Chi-squared, and Fisher’s exact tests.

### Sexual self-care outcomes

At baseline, no significant differences were observed between groups (p > 0.05). The intervention group demonstrated significant improvements across all sexual self-care domains compared to the control group (p < 0.05) (Table 4).

**Table 4:** Sexual self-care outcomes: Mean ± SD, between-group comparisons, 95% CI, and Cohen’s d.

Domain	Group	Baseline	Immediately After	8 Weeks After	Mean Difference (95% CI)	p-value
Prevention of STDs and Genital Infections	Intervention	70.36 ± 14.09	90.92 ± 7.91	95.36 ± 5.24	22.3 (16.1, 28.5)	<0.001
	Control	67.55 ± 10.48	68.94 ± 9.86	69.78 ± 10.36	–	0.072
p-value		0.342	< 0.001	< 0.001		
Prevention of Gynecological Cancers	Intervention	69.75 ± 17.65	86.10 ± 10.77	88.71 ± 10.39	21.1 (14.9, 27.3)	<0.001
	Control	65.41 ± 22.78	66.26 ± 18.59	66.65 ± 18.69	–	0.081
p-value		0.268	< 0.001	< 0.001		
Prevention of Unwanted Pregnancy	Intervention	69.48 ± 16.28	89.07 ± 9.33	93.01 ± 8.27	22.1 (15.8, 28.4)	<0.001
	Control	68.57 ± 13.18	69.16 ± 11.44	71.19 ± 10.52	–	0.193
p-value		0.796	< 0.001	< 0.001		
Promotion of Sexual Health	Intervention	62.83 ± 17.15	83.52 ± 14.98	86.64 ± 15.25	24.8 (18.2, 31.4)	<0.001
	Control	57.80 ± 17.98	60.27 ± 15.83	61.48 ± 17.16	–	0.09
p-value		0.228	< 0.001	< 0.001		
Overall Sexual Self-Care	Intervention	67.85 ± 12.13	87.39 ± 8.30	91.01 ± 8.29	23.1 (17.6, 28.6)	<0.001
	Control	64.69 ± 12.18	65.39 ± 9.87	65.98 ± 10.21	–	0.083
p-value		0.275	< 0.001	< 0.001		

Within-group changes over time were analyzed using Repeated Measures ANOVA, and between-group comparisons at each time point were performed using Independent t-tests.

### Health Belief Model (HBM) constructs

The intervention group showed significant improvements in perceived susceptibility, perceived benefits, cues to action, and self-efficacy, while perceived barriers decreased (p < 0.05). No significant changes were observed for perceived severity (p > 0.05). Interpretation of secondary outcomes considers potential Type I error due to multiple testing (Table 5).

**Table 5:** HBM constructs: Mean ± SD, between-group comparisons, 95% CI, and Cohen’s d.

Construct	Group	Baseline	Immediately After	8 Weeks After	Mean Difference (95% CI)	p-value
Perceived Susceptibility	Intervention	15.64 ± 3.16	18.11 ± 2.03	18.59 ± 1.77	4.3 (3.2, 5.4)	<0.001
	Control	14.62 ± 3.13	13.97 ± 3.39	13.94 ± 3.46	–	0.168
p-value		0.174	< 0.001	< 0.001		
Perceived Severity	Intervention	11.48 ± 4.07	10.86 ± 4.13	10.83 ± 4.26	0.3 (−0.6, 1.2)	0.223
	Control	11.88 ± 3.5	12.05 ± 3.07	11.94 ± 3.28	–	0.923
p-value		0.658	0.171	0.224		
Perceived Benefits	Intervention	19.89 ± 3.97	24.08 ± 1.27	24.32 ± 1.37	5.0 (4.1, 5.9)	<0.001
	Control	19.31 ± 4.01	18.97 ± 3.83	19.28 ± 3.69	–	0.512
p-value		0.541	< 0.001	< 0.001		
Perceived Barriers	Intervention	26.72 ± 6.09	25.29 ± 4.78	24.59 ± 5.14	−2.8 (−4.2, −1.4)	<0.001
	Control	27.34 ± 5.73	28.11 ± 5.54	28.22 ± 5.48	–	0.115
p-value		0.662	0.011	< 0.001		
Cues to Action	Intervention	11.54 ± 1.95	14.27 ± 1.04	14.67 ± 0.78	3.4 (2.7, 4.1)	<0.001
	Control	11.17 ± 2.75	11.05 ± 2.14	11.45 ± 2.04	–	0.492
p-value		0.513	< 0.001	< 0.001		
Self-Efficacy	Intervention	2.86 ± 0.91	4.08 ± 0.72	4.62 ± 0.54	1.7 (1.3, 2.1)	<0.001
	Control	2.82 ± 0.78	2.88 ± 0.71	3.05 ± 0.76	–	0.219
p-value		0.858	< 0.001	< 0.001		

Within-group changes over time were analyzed using Repeated Measures ANOVA, and between-group comparisons at each time point were performed using Independent t-tests.

Effect estimates were calculated using linear mixed-effects models; missing data (<5%) were handled with complete-case analysis; secondary outcomes are interpreted cautiously in light of multiple testing.

## Discussion

The results of this randomized controlled trial showed that an educational intervention based on the Health Belief Model (HBM) significantly enhanced sexual self-care practices among reproductive-age women with HPV. Notable improvements were found in areas such as the prevention of sexually transmitted infections, gynecologic cancer prevention, unintended pregnancy prevention, and the promotion of sexual health. Moreover, essential HBM constructs including perceived susceptibility, perceived benefits, cues to action, and self-efficacy improved, while perceived barriers were reduced.

Although most HBM constructs showed positive changes, perceived severity did not improve significantly. This outcome may indicate the difficulty of altering participants’ perception of the seriousness of a disease within a limited number of sessions. The second session of the intervention aimed to influence perceived susceptibility, severity, and benefits; however, reinforcing perceived severity may need a more direct focus. For example, explicitly highlighting the connection between HPV infection and specific cancers such as cervical, anal, and oropharyngeal cancers, supported by real-world statistics or examples, could strengthen participants’ awareness of possible consequences. Interactive strategies like scenario-based discussions or patient testimonials may further reinforce this aspect. Previous studies have also noted that perceived severity is less responsive to short-term educational interventions [15], emphasizing the need for more targeted approaches in this area.

These findings align with prior research that applied HBM to guide women’s health interventions. Bayrami et al. (2020) found that HBM constructs strongly predicted intentions to receive HPV vaccination among female students [16]. Similarly, Zandi et al. (2023) observed improvements in reproductive health behaviors among women with endometriosis following theory-driven education [21]. Masoudiyekta et al. (2018) reported significant increases in self-efficacy, perceived susceptibility, and perceived benefits in breast cancer screening programs, while perceived severity remained largely unchanged [24]. Cao et al. (2014) also highlighted that perceived benefits and severity played crucial roles in injury prevention education among adolescents, though perceived barriers had less influence [25]. Together, these findings across diverse health areas reinforce the robustness of the HBM framework.

In contrast, some studies have shown inconsistent results regarding the predictive strength of certain HBM constructs. Carpenter’s (2010) meta-analysis concluded that perceived severity had relatively weak predictive power, consistent with the lack of significant change observed in this study [15].

Our results also contribute to filling knowledge gaps regarding HPV and sexual health documented in prior surveys. For instance, Pourkazemi et al. (2013) reported that only a small percentage of Iranian women were sufficiently aware of HPV transmission and prevention [26], while Gerend and Magloire (2008) found that many U.S. college women underestimated their susceptibility to HPV [27]. McBride et al. (2021) further noted that stigma and anxiety acted as barriers to care-seeking among women with HPV [28]. The current trial demonstrates that a structured, theory-based intervention can translate knowledge into observable behavioral improvements. Large effect sizes (Cohen’s d > 1.2 across most domains) suggest that bridging knowledge gaps through targeted education not only raises awareness but also boosts motivation and self-efficacy, encouraging sustained self-care behaviors.

Beyond enhancing sexual self-care, the HBM-based questionnaire used in this trial may serve as a useful tool in clinical counseling. By systematically evaluating patients’ beliefs, perceived barriers, and readiness for behavior change, healthcare providers can better customize counseling, thereby improving intervention outcomes.

### Strengths and limitations

The strengths of this study include employing HBM as a strong theoretical foundation, utilizing a randomized controlled trial design, and applying validated tools such as the FSHS questionnaire. The use of multimedia-based and interactive teaching methods, along with reporting effect sizes and confidence intervals, added to the methodological rigor.

However, limitations include the inability to blind participants and researchers, the short follow-up duration of eight weeks, and recruitment restricted to two hospitals in Tehran, which may limit the generalizability of the findings. Additionally, cultural and social dynamics unique to the Iranian context may affect the external validity.

### Recommendations

Future research should involve larger, multicenter, or nationwide samples to improve representativeness. Extending follow-up durations is recommended to assess the sustainability of behavior changes. Comparative studies that test alternative theoretical models like the Theory of Planned Behavior or Social Cognitive Theory may reveal the most effective approach. Interdisciplinary educational programs involving gynecologists, midwives, psychologists, and health educators should be explored to address the multifaceted nature of sexual health. Incorporating digital health solutions such as mobile apps, online learning modules, and telehealth counseling could expand accessibility and continuity of care. Tailoring the educational material to sociocultural contexts and involving partners where relevant may further enhance outcomes.

## Conclusion

This study confirms that HBM-based educational interventions can significantly improve sexual self-care behaviors among HPV-positive women. Improvements in perceived susceptibility, perceived benefits, self-efficacy, and cues to action indicate the utility of HBM in designing effective preventive interventions. By reducing barriers and empowering women with knowledge and confidence, such interventions can mitigate adverse HPV outcomes and strengthen reproductive health. HBM-based educational programs should be integrated into public health policy and clinical practice, especially for vulnerable populations.

## Supporting information

S1 File Protocol. (DOCX)

## Abbreviations

HPV: human papillomavirus
HBM: Health Belief Model
FSHS: The Sexual Self-Care in Women of Reproductive Age questionnaire
CVI: content validity index
CVR: content validity ratio.
