# Supplementary material for: Empowering sexual self-care in HPV-positive women: A randomized trial of health belief model–Based education
Source: PLoS One. 2025 Dec 11;20(12):e0338192. doi: 10.1371/journal.pone.0338192 (PMC12697967; doi:10.1371/journal.pone.0338192)
Supplement: S1 File — (DOCX) [file pone.0338192.s001.docx]

**Improving** **Sexual Self-Care Among Women Infected with** **Human Papillomavirus Through an Educational Intervention Based on the** **Health Belief Model: A Randomized Controlled Trial Protocol**

Sanaz Akhondzadeh^1^, Zahra Behboodi Moghadam^2^, Masoumeh Namazi^*3^

1. M.S. of midwifery, Department of midwifery and reproductive health, School of nursing and midwifery, Tehran University of Medical Sciences, Tehran, Iran.

e-mail: sanaz9924a@gmail.com

Tel: +989357116428

1. Professor, Department of midwifery and reproductive health, School of nursing and midwifery, Tehran University of Medical Sciences, Tehran, Iran.

e-mail: Behboodi@tums.ac.ir

Tel: +989122494201

1. Corresponding Author, Assistant professor, Department of midwifery and reproductive health, School of nursing and midwifery, Tehran University of Medical Sciences, Tehran, Iran.

e-mail: masnamazi@yahoo.com, mnamazi@sina.tums.ac.ir

Tel: +989188116771

*Corresponding author: Masoumeh Namazi, Ph.D. of Reproductive Health, Assistant Professor, Department of Midwifery and Reproductive Health, School of Nursing and Midwifery, Tehran University of Medical Sciences, Tehran, Iran, Tel: +982161054221, Fax: +982166904252, P.O. Box: +981419733171, Email: [masnamazi@yahoo.com](mailto:masnamazi@yahoo.com), mnamazi@sina.tums.ac.ir

**Abstract**

**Background:** The human papillomavirus (HPV) is the most common sexually transmitted infection worldwide. The present study aims to examine the impact of an educational intervention based on the Health Belief Model (HBM) on sexual self-care among women of reproductive age infected with HPV.

**Method:** This is a randomized controlled trial with two parallel groups. Seventy women with HPV will be selected through simple random sampling from Arash Women's Comprehensive Hospital and Imam Khomeini Hospital in Tehran, Iran and will be allocated to a control group (n = 35) and an intervention group (n = 35). Participants in the intervention group will receive HBM-based sexual self-care education in four 45-60 min weekly sessions which will be held using the lectures, group discussions, question & answer sessions, PowerPoint presentations, pamphlets, educational videos, and role-playing. Data will be collected through a Demographic and obstetric information questionnaire, The Sexual Self-Care and HBM questionnaires, and will be analyzed through the ANOVA, Friedman test, and logistic regression modeling. All statistical analyses will be performed using SPSS version 24.

**Discussion:** Upon completion of this study, an increase in sexual self-care scores may indicate the effectiveness of this educational intervention. If proven effective, it can be recommended as a cost-effective method to achieve this goal, and it can be utilized by health professionals, including midwives and counselors to enhance sexual self-care among these women.

**Key-words:** Sexual Self-Care, Human Papillomavirus, Women, Health Belief Model, Intervention, Clinical trial

**Ethical code:** IR.TUMS.FNM.REC.1402.186

**Trial registration:** This study was registered in the Iranian Registry of Clinical Trials on 2024/01/01 (code: IRCT20231223060503N1)

**Plain English summary**

Human papillomavirus (HPV) is the most common sexually transmitted infection worldwide. Most HPV-related diseases are preventable through education, screening programs, and vaccination. However, many people lack sufficient information about prevention, screening, and self-care. This research will be an interventional study aimed at assessing the impact of an educational method on the sexual self-care of women with HPV. We will have two groups of women with HPV, each containing 35 participants. One group will participate in an educational program on sexual self-care, consisting of four sessions that include lectures, group discussions, Q&A sessions, PowerPoint presentations, pamphlets, educational videos, and role-playing. The other group will receive routine care. Finally, we will assess the effectiveness of the education on the sexual self-care of women with HPV.

**Background**

The human papillomavirus (HPV) is the most common sexually transmitted infection worldwide [1]. HPV strains are classified into low-risk and high-risk types based on their association with cancer [2]. The global prevalence of HPV is estimated to be 11.7%, with the highest rates observed in Southern Africa (17.4%), Eastern Africa (33.6%), Eastern Europe (21.4%), and Western Europe (9.0%) [3]. HPV transmission occurs through direct skin-to-skin or mucosal contact during vaginal, anal, or oral sex. Both asymptomatic and symptomatic individuals infected with HPV can transmit the virus [4]. However, most anogenital HPV infections resolve on their own; for example, over 90% of cervical infections clear within 1 to 3 years [5]. High-risk sexual behaviors, including a young age at first vaginal or oral intercourse and having more sexual partners, are the main risk factors associated with the acquisition and persistence of HPV infection and the development of related cancers. These sexual risk factors vary based on socioeconomic status, age, race, and education level [6,7]. It is estimated that around 2.8% of Iranian women in the general population have HPV16/18 infection. Iran has a population of about 33.5 million women aged 15 years and older at risk of developing cervical cancer, with 1,056 women diagnosed with cervical cancer and 644 deaths annually [8].

Most HPV-related diseases are preventable through education, screening programs, and vaccination [1]. HPV infection and genital warts have significant physical and psychological effects on women [9]. The more severe the disease, the greater the impact on mental health, which also imposes financial costs on healthcare systems [10]. A lack of awareness about safe sexual practices, the protective benefits of HPV vaccination, and the need for a range of tests could be the primary reasons for these issues [11].

Self-care refers to the ability of individuals, families, and communities to take intentional and purposeful actions to promote and maintain their health, prevent illness, and manage disease and disability, with or without the support of healthcare providers [12]. Effective techniques for enhancing self-care behaviors in women are organized into three categories: lifestyle modification, preventive behaviors and screening, and disease and treatment management behaviors. Self-care behaviors that help prevent HPV infection include undergoing Pap smears, vaccination, consistent and correct use of condoms during sexual intercourse, and limiting sexual partners to one [11,12]. Self-care interventions are among the most important and promising approaches to improving universal health coverage and well-being, both for health systems and individuals. As accessible, cost-effective, and acceptable interventions, they promote self-efficacy, independence, and participation in health [13], yet nearly four billion women of reproductive age worldwide lack access to such services [14].

One of the most widely used theories in health behavior is the Health Belief Model (HBM), which has extensive applications in health education for various health-related issues. The HBM posits that six constructs predict health behavior: perceived susceptibility, perceived severity, perceived benefits, perceived barriers, self-efficacy, and cues to action [15]. A study by Bayrami et al. (2019) aimed to assess the constructs of the HBM in relation to the willingness to receive HPV vaccination among female students at Urmia University of Medical Sciences. According to the results, perceived susceptibility, perceived severity, perceived benefits, perceived barriers, self-efficacy, and cues to action were all significantly associated with the acceptance of the HPV vaccine [16].

According to the results of a qualitative study conducted in Iran (2023), most women infected with HPV lacked sufficient knowledge about the virus, including its causes, symptoms, complications, prevention, and screening methods. Education, counseling, support, and healthcare services were identified as the main needs and challenges in reproductive and sexual health [17]. Based on the literature review, most studies have focused on the prevention of HPV infection. However, individuals who are already affected by this condition face numerous challenges, highlighting the need for further research into self-care practices for this group of patients. Given the Iranian and Islamic cultural context, HPV infection may be associated with negative psychological effects and significant challenges for Iranian women due to its sexual nature and the stigma attached to it, complicating efforts to educate, support, and address their needs [18,19]. Therefore, the present study aims to examine the impact of an educational intervention based on the Health Belief Model on sexual self-care among women of reproductive age infected with HPV.

**Hypothesis:** The educational intervention based on the Health Belief Model will lead to changes in perceived susceptibility, severity, benefits, barriers, and self-efficacy among women with HPV. These changes will, in turn, modify individual behavior and enhance sexual self-care in those affected by this disease.

**Objectives:** The primary objective of this study is to evaluate the effectiveness of an educational intervention based on the Health Belief Model on sexual self-care among women infected with the HPV. The secondary objectives of the study are:

1. To determine and compare the sexual self-care scores among women with HPV in the intervention and control groups before, immediately after, and 8 weeks following the intervention.
2. To determine and compare the items of the Health Belief Model (perceived susceptibility, perceived severity, perceived benefits, perceived barriers, cues to action, and self-efficacy) regarding sexual self-care among women with HPV in the intervention and control groups before, immediately after, and 8 weeks following the intervention.

**Method**

***Design***

This study will be conducted as a randomized clinical trial with one intervention group and one control group. The study aims to provide education based on the Health Belief Model to improve sexual self-care among women with HPV who attend the gynecology clinics of hospitals affiliated with Tehran University of Medical Sciences. This trial will follow the CONSORT guidelines (Figure 1) [20]. The current protocol has been structured according to the “Standard Protocol Items: Recommendations for Interventional Trials” (SPIRIT) checklist [21]. This study has been approved by the Ethics Committee of Tehran University of Medical Sciences with the ethics code (IR.TUMS.FNM.REC.1402.186) and has been registered as a clinical trial with the code IRCT20231223060503N1.

Figure 1. Participants’ flow in the study

## Follow-Up

Analysed (n= )
♦ Excluded from analysis (give reasons) (n= )

## Analysis

Analysed (n= )
♦ Excluded from analysis (give reasons) (n= )

Lost to follow-up (give reasons) (n= )

Discontinued intervention (give reasons) (n= )

Lost to follow-up (give reasons) (n= )

Discontinued intervention (give reasons) (n= )

## Enrollment

Allocated to intervention (n=35)

♦ Received allocated intervention (n= )

♦ Did not receive allocated intervention (give reasons) (n= )

## Allocation

Allocated to intervention (n=35)

♦ Received allocated intervention (n= )

♦ Did not receive allocated intervention (give reasons) (n= )

Randomized (n=70)

Excluded (n= )

♦  Not meeting inclusion criteria (n= )

♦  Declined to participate (n= )

♦  Other reasons (n= )

Assessed for eligibility (n= )

***Study population***

Study population consists of all women with HPV

***Study setting***

The setting of the study will be Arash Women's Comprehensive Hospital and Imam Khomeini Hospital. These clinics have been selected as referral hospitals in central and eastern Tehran, Iran.

***Participants***

Participants will be women with HPV who visit the women’s clinics at Arash and Imam Khomeini hospitals and meet the study inclusion criteria.

***Inclusion Criteria***

- - Positive HPV-DNA test
  - Iranian nationality
  - Married
  - Age between 15 and 49 years
  - Literacy (ability to read and write)
  - No chronic psychiatric disorders
  - No known malignant diseases
  - No previous participation in educational or counseling sessions related to STIs

***Exclusion Criteria***

- - Absence from more than two educational sessions
  - Incomplete or incorrect completion of the questionnaire
  - Lack of willingness to continue participating in the study

***Sampling method***

After the study proposal is approved by the Ethics Committee of Tehran University of Medical Sciences, and after obtaining a Clinical Trial Registration Code (IRCT) and a written letter of introduction, eligible women will be enrolled in the study. Ethical conditions and considerations will be explained, and written informed consent will be obtained from those willing to participate. The researcher will first provide detailed information about the study, including its title, objectives, inclusion criteria, and the study's procedures. Participants will then be randomly assigned to either the intervention or control group using block randomization. Specifically, volunteers who agree to participate will be randomly allocated to the intervention or control groups using a block size of four. The educational intervention will be conducted in person. The intervention group will receive training based on the Health Belief Model, focusing on its constructs, in four sessions of 45-60 minutes each, held once a week with groups of 10-15 participants. This training will be provided by the researcher. The control group will receive routine care. The questionnaires will be administered to participants in both groups at three time points: before the intervention, immediately after, and 8 weeks post-intervention. To adhere to ethical considerations, the control group, if interested in receiving the training, will be provided with educational pamphlets and CDs after the intervention is completed. Figure 2 shows the schedule of enrolment, intervention, and assessment.

Figure 2. Schedule of enrolment, interventions, and assessments

|  | **STUDY PERIOD** | | | | | | | |  |
| --- | --- | --- | --- | --- | --- | --- | --- | --- | --- |
|  | **Enrolment** | **Allocation** | **Post-allocation** | | | | **Close-out** | |  |
| **TIMEPOINT**** | ***-t_1_*** | **0** | ***t_1_*** | ***t_2_*** | ***t_3_*** | ***t_4_*** | | ***t_5_*** | |
| **ENROLMENT:** |  |  |  |  |  |  | |  | |
| **Eligibility screen** | X |  |  |  |  |  | |  | |
| **Informed consent** | X |  |  |  |  |  | |  | |
| ***Randomized by independent person*** | X |  |  |  |  |  | |  | |
| **Allocation** |  | X |  |  |  |  | |  | |
| **INTERVENTIONS:** |  |  |  |  |  |  | |  | |
| ***Group A: educational intervention*** |  |  |  |  |  |  | |  | |
| ***Group B: control*** |  |  |  |  |  |  | |  | |
| **ASSESSMENTS:** |  |  |  |  |  |  | |  | |
| ***Demographic and obstetric information questionnaire*** | X |  |  |  |  |  | |  | |
| ***FSHS*** | X |  |  |  |  |  | | X | |
| ***Researcher-Designed Questionnaire Based on the HBM*** | X |  |  |  |  |  | | X | |

***Sample Size***

To determine the necessary sample size, with a confidence level of 95%, a test power of 80%, and assuming that the effect size (Cohen's d) of the intervention compared to the control group is 0.7 [22,23], the minimum sample size required for each group is calculated to be 32 participants. Considering a 10% dropout rate, the sample size for each group will be set at 35 participants.

n = (2 × (z _(1-α/2)_ + z _(1-β)_))^2^ / (E × S)^2^ = (2 × (1.96 + 0.84))^2^ / (0.7)^2^ = 32

z _(0.975)_ = 1.96

z _(0.8)_ = 0.84

***Randomization and allocation***

Participants will be randomly assigned to either the intervention or control group using a block randomization method with a block size of four. The letter A represents the intervention group, and the letter B represents the control group. All possible permutations of the letters AA and BB will be listed, resulting in six different combinations (AABB, BBAA, ABAB, BAAB, ABBA, BBBA). One combination will be randomly selected from these six permutations. For example, if the combination AABB is chosen, it means that the first and second participants will be assigned to the intervention group, and the next two participants will be assigned to the control group. This process will continue until the sample size is reached.

***Allocation concealment and blinding***

To ensure allocation concealment, a colleague not involved in the study will create the allocation sequence and write it on pieces of paper. These papers will be placed in opaque envelopes, which will then be sequentially numbered. The envelopes will be opened in order to assign participants to their respective groups. Although blinding of researchers and participants is not feasible due to the nature of the intervention, the outcome evaluator will remain blinded by having a separate colleague handle data collection.

***Outcomes***

The primary outcome of the study is sexual self-care which will be measured through the sexual self-care in women of reproductive age (FSHS) questionnaire. The secondary outcome of the study will be participants’ perceived Susceptibility, severity, benefits, barriers, and self-efficacy regarding sexual self-care which will be measured through the researcher-designed questionnaire based on the HBM.

***Educational Intervention***

The constructs of the HBM are outlined as follows (Table 1):

1. **Perceived Susceptibility:** This refers to an individual's belief about the likelihood of experiencing a health threat or developing a health problem. Since infection with human papillomavirus (HPV) can affect individuals' functioning, assessing perceived susceptibility and providing the necessary education can bring about changes in this construct.
2. **Perceived Severity:** This construct addresses the seriousness, severity, and gravity of the disease or problem. Individuals’ understanding of the issues related to being infected with HPV and the resulting complications varies. In this dimension, the extent of problems and symptoms will be identified.
3. **Perceived Benefits:** When an individual acknowledges that they might suffer from the complications of this infection, they will adopt the suggested behavior and engage in actions that provide the most benefit, which encourages consistent participation in educational sessions.
4. **Perceived Barriers:** This construct encompasses the negative aspects that discourage a person from engaging in the recommended behavior. These negative aspects may include the costs involved, discomfort, inconvenience, time consumption, perceived inappropriateness, futility, and lack of self-esteem and dignity.
5. **Self-Efficacy:** An individual infected with HPV faces multiple challenges and complications. Overcoming these problems is a crucial factor in the continuation of the health behavior taught. Since acting based on an individual's incorrect judgment of their personal abilities can lead to undesirable outcomes, an accurate assessment of personal abilities is of high practical value and leads to self-efficacy in behavior.

**Table 1. Health Belief Model-Based Educational Intervention**

| **Session** | **Main Objective** | **Educational Content** | **Teaching Methods and Schedule** |
| --- | --- | --- | --- |
| First | Improve awareness about HPV and sexual self-care | PowerPoint presentation, Session 1 handout | 1) Introduction of the facilitator and participants, study objectives (10 min) 2) Information on HPV, its global and local prevalence, and the importance of sexual self-care (40 min) 3) Q&A session (10 min) |
| Second | Impact on perceived susceptibility, perceived severity, and perceived benefits of the Health Belief Model | PowerPoint presentation, Session 2 handout | 1) Review of the previous session (5 min) 2) Explanation of the consequences of not having a Pap smear test, non-vaccination against non-infecting strains, effects on marital life and quality of life, issues with having multiple sexual partners (45 min) 3) Q&A session to address perceived benefits (10 min) |
| Third | Impact on perceived barriers and self-efficacy of the Health Belief Model | PowerPoint presentation, Session 3 handout, Educational video | 1) Review of previous sessions (5 min) 2) Request women with HPV to share their issues since the onset of symptoms or diagnosis, discuss the concept of sexual self-care and its connection to self-efficacy (40 min) 3) Share common experiences and solutions with each other, summarize the taught materials using the educational video (15 min) 4) Q&A session (10 min) |
| Fourth | Impact on cues to action and performance of the Health Belief Model | PowerPoint presentation, Session 4 handout, Educational video, Role-playing | 1) Review of previous sessions (5 min) 2) Teach various sources of information about HPV (15 min) 3) Ask women to express their fears about symptoms and provide self-care recommendations, advise appropriate behaviors, and communicate problems and needs to healthcare providers (30 min) 4) Role-playing, educational video, and summarization of the taught materials (10 min) 5) Q&A session (10 min) |

***Intervention Design***

The educational intervention will be conducted by the researcher in 4 in-person sessions, each lasting 45-60 minutes, once a week with groups of 10-15 participants. The intervention will be based on the Health Belief Model. Educational methods in this study will include lectures, group discussions, Q&A sessions, PowerPoint presentations, pamphlets, educational videos, and role-playing. The control group will receive routine care. The Sexual Self-Care and HBM questionnaires will be administered to participants in both groups at three points: before the intervention, immediately after, and 8 weeks following the completion of the intervention. To address ethical considerations, the control group participants who wish to receive the education will be provided with pamphlets and educational CDs after the intervention is completed.

***Study instruments***

The three study instruments are explained in what follows.

**-****Demographic and obstetric information questionnaire**: It consists of 16 questions including: Age, Spouse's Age, Education Level, Spouse's Education Level, Employment Status, Spouse's Occupation, Income Level, Duration of Marriage, Number of Pregnancies, Number of Deliveries, Type of Delivery, Number of Abortions, Contraceptive Method, History of Pap Smear, History of HPV Vaccination, Duration of HPV Infection Based on Diagnostic Tests.

-**The Sexual Self-Care in Women of Reproductive Age (****FSHS) questionnaire**: It consists of 40 items scored on a 5-point Likert scale ranging from Never (1) to Always (5) across 4 domains: prevention of sexually transmitted infections (STIs), prevention of gynecological cancers, prevention of unintended pregnancies, and promotion of sexual health. Developed using a deductive approach based on the Waltz model in 2021 by Yazdani and colleagues in Iran, the tool has demonstrated a content validity index (CVI) of 0.93, a content validity ratio (CVR) of 0.96, and a high internal consistency reliability with a Cronbach's alpha of 0.94. The stability of the instrument, measured by intra-cluster correlation, was 0.97 (confidence interval: 0.94-0.98), indicating strong validity and reliability in the Iranian population, making it a suitable measure of sexual self-care [24].

**-Researcher-Designed Questionnaire Based on the Health Belief Model**: This questionnaire is designed by researchers based on the study objectives and relevant literature. The questions are shown in Table 2. To confirm the qualitative content validity of the questionnaire, it will be reviewed by 10 faculty members in midwifery and reproductive health at Tehran University of Medical Sciences, and their feedback will be incorporated. For the CVR, experts will be asked to evaluate each item on a 3-point scale (essential, useful but not essential, not essential). After calculating the CVR and making necessary revisions, experts will also assess the CVI by rating each item on a scale from 1 to 4 based on relevance (4 = very relevant, 3 = relevant, 2 = somewhat relevant, 1 = not relevant). Face validity will be assessed by asking 10 women of reproductive age to provide feedback on the clarity of the questionnaire, and necessary clarifications will be made based on their input. Internal consistency will be evaluated using Cronbach's alpha to determine the reliability of the instrument.

**Table 2. Researcher-Designed Questionnaire Based on the Health Belief Model**

| **Question Type** | **Questions** | **Scale** |
| --- | --- | --- |
| Perceived Susceptibility | 1. I am at risk of complications from HPV. 2. I am at risk of cervical cancer. 3. I am at risk of high-risk HPV strains. 4. My partner is at risk of the disease. | 5-point Likert scale (1=Strongly disagree, 5=Strongly agree) |
| Perceived Severity | 1. The disease causes problems in sexual relations. 2. Thinking about the complications of HPV scares me. 3. Thinking about the problems caused by HPV infection makes me anxious and worried. 4. If I experience HPV complications, my spouse and family will blame me. 5. The cost of treatment is high. 6. Quality of life decreases after contracting the disease. | 5-point Likert scale (1=Strongly disagree, 5=Strongly agree) |
| Perceived Benefits | 1. Women who engage in sexual self-care experience fewer physical and emotional crises. 2. Sexual self-care reduces the costs associated with cervical cancer. 3. Sexual self-care prevents my partner from contracting the disease. 4. Sexual self-care allows me to enjoy sexual relations more. 5. Sexual self-care helps prevent high-risk HPV strains. | 5-point Likert scale (1=Strongly disagree, 5=Strongly agree) |
| Perceived Barriers | 1. I don't have enough time to get the HPV vaccine. 2. I don't have enough time to visit a doctor or midwife. 3. I can't afford the HPV vaccine. 4. I don't have access to accurate information about sexual self-care. 5. My partner agrees to use condoms during sexual relations. 6. I feel embarrassed about vaginal exams and Pap smears. 7. I am afraid of vaginal exams and Pap smears. 8. Fear of side effects from the HPV vaccine prevents me from getting it. | 5-point Likert scale (1=Strongly disagree, 5=Strongly agree) |
| Self-Efficacy | 1. Reading brochures and using educational videos are effective for sexual self-care. 2. Media (TV and social media) are effective for sexual self-care. 3. My doctor and midwife assist me with sexual self-care. | 5-point Likert scale (1=Strongly disagree, 5=Strongly agree) |

***Data management and analysis***

Descriptive analysis will be used to report baseline characteristics. For continuous variables with a normal distribution, ANOVA will be used between subjects, and for continuous variables without a normal distribution, the Friedman test will be employed. The effectiveness of the intervention will be evaluated using ANOVA, and the impact of demographic variables on sexual self-care will be determined using logistic regression modeling. All statistical analyses will be performed using SPSS version 24. All statistical analyses will be performed using the per protocol analysis and at a significance level of less than 0.05.

**Discussion**

This randomized controlled trial will assess the impact of an educational intervention based on the HBM on sexual self-care among women of reproductive age infected with HPV.

Most HPV-related diseases can be prevented through education, screening programs, and vaccination [1]. Therefore, a well-designed, targeted educational program is crucial for promoting and maintaining sexual self-care among women, preventing disease, and managing illness and disability. Given the Iranian and Islamic cultural context, women affected by HPV may face significant psychological challenges and stigma due to the sexual nature of the virus, which can hinder their ability to receive education, support, and appropriate care [18, 19]. A qualitative study in Iran (2023) found that most women with HPV lacked sufficient knowledge about the virus, its causes, symptoms, complications, prevention, and screening methods. Moreover, education, counseling, support, and healthcare services were identified as key needs and challenges in reproductive and sexual health [17].

Upon completion of this study, an increase in sexual self-care scores may indicate the effectiveness of this educational intervention. If proven effective, it will contribute to the development of programs aimed at improving sexual self-care. HBM-based education can be recommended as a cost-effective method to achieve this goal, and it can be utilized by health professionals, including midwives and counselors, to enhance sexual self-care among these women. Conversely, if the results are not as expected, they could guide us in designing new strategies to improve sexual self-care. Additionally, this study will assess which HBM construct is the most significant and impactful in promoting sexual self-care, providing insights that can inform the content used in future programs and research.

**List of abbreviations**

HPV: human papillomavirus

HBM: Health Belief Model

SPIRIT: Standard Protocol Items: Recommendations for Interventional Trials

FSHS: The Sexual Self-Care in Women of Reproductive Age questionnaire

CVI: content validity index

CVR: content validity ratio

**Declarations**

**Ethics approval and consent to participate**

Written informed consent will be obtained from each participant. Additionally, this protocol was approved by the Ethics Committee of Tehran University of Medical Sciences, Tehran, Iran IR.TUMS.FNM.REC.1402.186.

**Consent for publication**

Not applicable.

**Availability of data and materials**

Not applicable.

**Competing interests**

There are no conflicts of interest.

**Funding**

This study is funded by the Tehran University of Medical Sciences.

**Authors**' **contributions**

ZBM, and MN contributed to the design of the protocol. MN, and SA contributed to the implementation and analysis plan. MN and SA wrote the first draft of this protocol article. All authors have critically read the text and contributed inputs and revisions, and all authors have read and approved the final manuscript.

**Acknowledgments**

We would like to thank Vice-Chancellor for Research at Tehran University of Medical Sciences for their financial support.

**References**

1. Plotzker RE, Vaidya A, Pokharel U, Stier EA. Sexually transmitted human papillomavirus: update in epidemiology, prevention, and management. Infectious Disease Clinics. 2023;37(2):289-310.
2. Doorbar J, Egawa N, Griffin H, Kranjec C, Murakami I. Human papillomavirus molecular biology and disease association. Reviews in medical virology. 2015;25:2-3.
3. Soheili M, Keyvani H, Soheili M, Nasseri S. Human papilloma virus: A review study of epidemiology, carcinogenesis, diagnostic methods, and treatment of all HPV-related cancers. Medical journal of the Islamic Republic of Iran. 2021;35:65.
4. Castellsagué X. Natural history and epidemiology of HPV infection and cervical cancer. Gynecologic oncology. 2008;110(3):S4-7.
5. Burd EM. Human papillomavirus and cervical cancer. Clinical microbiology reviews. 2003;16(1):1-7.
6. Rettig E, Kiess AP, Fakhry C. The role of sexual behavior in head and neck cancer: implications for prevention and therapy. Expert review of anticancer therapy. 2015;15(1):35-49.
7. Halpern CT, Haydon AA. Sexual timetables for oral-genital, vaginal, and anal intercourse: Sociodemographic comparisons in a nationally representative sample of adolescents. American Journal of Public Health. 2012;102(6):1221-8.
8. ICO/IARC Information Centre on HPV and Cancer, Human Papillomavirus and Related Cancers, Fact Sheet 2023 Iran [www.hpvcentre.net](http://www.hpvcentre.net)
9. Escalas J, Rodriguez-Cerdeir C, Guerra-Tapia A. Impact of HPV infection on the quality of life in young women. The Open Dermatology Journal. 2009;3(1).
10. McBride E, Tatar O, Rosberger Z, Rockliffe L, Marlow LA, Moss-Morris R, Kaur N, Wade K, Waller J. Emotional response to testing positive for human papillomavirus at cervical cancer screening: a mixed method systematic review with meta-analysis. Health psychology review. 2021;15(3):395-429.
11. Sikorska M, Pawłowska A, Antosik-Wójcińska A, Zyguła A, Suchońska B, Dominiak M. The Impact of HPV Diagnosis and the Electrosurgical Excision Procedure (LEEP) on Mental Health and Sexual Functioning: A Systematic Review. Cancers (Basel). 2023;15(8):2226.
12. Yari F, Moghadam ZB, Parvizi S, Nayeri ND, Rezaei E, Saadat M. An evaluation of the effectiveness of a reproductive health education program for nonmedical students in Iran: A quasi-experimental pre-test, post-test research. Shiraz E-Medical Journal. 2016;17(3).
13. Narasimhan M, Hargreaves JR, Logie CH, Abdool-Karim Q, Aujla M, Hopkins J, Cover J, Sentumbwe-Mugisa O, Maleche A, Gilmore K. Self-care interventions for women’s health and well-being. Nature medicine. 2024;30(3):660-9.
14. Riegel B, Dunbar SB, Fitzsimons D, Freedland KE, Lee CS, Middleton S, Stromberg A, Vellone E, Webber DE, Jaarsma T. Self-care research: where are we now? Where are we going?. International journal of nursing studies. 2021;116:103402.
15. Carpenter CJ. A meta-analysis of the effectiveness of health belief model variables in predicting behavior. Health communication. 2010;25(8):661-9.
16. bayrami R, Didarloo A, Khalkhali H R, ayatollahi H, Ghorbani B. Relationship between health belief model and human papillomavirus vaccine intent among female students of Urmia university of medical sciences. Nursing and Midwifery Journal 2020; 17 (11) :897-906
17. Galeshi M, Shirafkan H, Yazdani S, Motaghi Z. Challenges and Needs of HPV-Positive Women. Inquiry. 2023; 60.
18. Nasirian M, Kianersi S, Hoseini SG, Kassaian N, Yaran M, Shoaei P, Ataei B, Fadaei R, Meshkati M, Naeini AE, Jalilian MR. Prevalence of sexually transmitted infections and their risk factors among female sex workers in Isfahan, Iran: a cross-sectional study. Journal of the International Association of Providers of AIDS Care (JIAPAC). 2017;16(6):608-14.
19. Ghorashi Z. Sexually transmitted infections in Iran: A literature review. Journal of Occupational Health and Epidemiology. 2015;4(4):260-5.
20. Schulz KF, Altman DG, Moher D. CONSORT 2010 statement: updated guidelines for reporting parallel group randomised trials. Journal of Pharmacology and pharmacotherapeutics. 2010;1(2):100-7.
21. Chan AW, Tetzlaff JM, Gøtzsche PC, Altman DG, Mann H, Berlin JA, Dickersin K, Hróbjartsson A, Schulz KF, Parulekar WR, Krleža-Jerić K. SPIRIT 2013 explanation and elaboration: guidance for protocols of clinical trials. Bmj. 2013;346.
22. Zandi N, Behboodi Moghadam Z, Hossein Rashidi B, Namazi M, Haghani S. Reproductive health of women with endometriosis: an improving educational intervention based on the planned behavior theory. Middle East Fertility Society Journal. 2023;28(1):4.
23. Allen Jr JC. Sample size calculation for two independent groups: A useful rule of thumb. Proceedings of Singapore Healthcare. 2011;20(2):138-40.
24. Yazdani F, Simbar M, Zare E, Hamzeh Gardeshi Z, Nasiri M. Design and psychometric of sexual self-care questionnaire in reproductive age women based on Waltz model. The Iranian Journal of Obstetrics, Gynecology and Infertility. 2023;25(12): 43-63

**Legends:**

Figure 1. Participants’ flow in the study

Figure 2. Schedule of enrolment, interventions, and assessments

Table 1. Health Belief Model-Based Educational Intervention

Table 2. Researcher-Designed Questionnaire Based on the Health Belief Model
